# Lung cells support osteosarcoma cell migration and survival

**DOI:** 10.1186/s12885-017-3047-5

**Published:** 2017-01-25

**Authors:** Shibing Yu, Mitchell Stephen Fourman, Adel Mahjoub, Jonathan Brendan Mandell, Jared Anthony Crasto, Nicholas Giuseppe Greco, Kurt Richard Weiss

**Affiliations:** 1 Department of Orthopaedic Surgery, University of Pittsburgh, Pittsburgh, PA USA; 2 School of Medicine, University of Pittsburgh, Pittsburgh, PA USA; 3 Cancer Stem Cell Laboratory, Department of Orthopaedic Surgery, 450 Technology Dr, Pittsburgh, PA 15219 USA

**Keywords:** Osteosarcoma, Metastasis, Lung microenvironment, Disulfiram, Aldehyde dehydrogenase

## Abstract

**Background:**

Osteosarcoma (OS) is the most common primary bone tumor, with a propensity to metastasize to the lungs. Five-year survival for metastatic OS is below 30%, and has not improved for several decades despite the introduction of multi-agent chemotherapy. Understanding OS cell migration to the lungs requires an evaluation of the lung microenvironment. Here we utilized an in vitro lung cell and OS cell co-culture model to explore the interactions between OS and lung cells, hypothesizing that lung cells would promote OS cell migration and survival. The impact of a novel anti-OS chemotherapy on OS migration and survival in the lung microenvironment was also examined.

**Methods:**

Three human OS cell lines (SJSA-1, Saos-2, U-2) and two human lung cell lines (HULEC-5a, MRC-5) were cultured according to American Type Culture Collection recommendations. Human lung cell lines were cultured in growth medium for 72 h to create conditioned media. OS proliferation was evaluated in lung co-culture and conditioned media microenvironment, with a murine fibroblast cell line (NIH-3 T3) in fresh growth medium as controls. Migration and invasion were measured using a real-time cell analysis system. Real-time PCR was utilized to probe for Aldehyde Dehydrogenase (ALDH1) expression. Osteosarcoma cells were also transduced with a lentivirus encoding for GFP to permit morphologic analysis with fluorescence microscopy. The anti-OS efficacy of Disulfiram, an ALDH-inhibitor previously shown to inhibit OS cell proliferation and metastasis in vitro, was evaluated in each microenvironment.

**Results:**

Lung-cell conditioned medium promoted osteosarcoma cell migration, with a significantly higher attractive effect on all three osteosarcoma cell lines compared to basic growth medium, 10% serum containing medium, and NIH-3 T3 conditioned medium (*p* <0.05). Lung cell conditioned medium induced cell morphologic changes, as demonstrated with GFP-labeled cells. OS cells cultured in lung cell conditioned medium had increased alkaline phosphatase staining.

**Conclusions:**

Lung endothelial HULEC-5a cells are attractants for OS cell migration, proliferation, and survival. The SJSA-1 osteosarcoma cell line demonstrated greater metastatic potential than Saos-2 and U-2 cells. ALDH appears to be involved in the interaction between lung and OS cells, and ALP may be a valuable biomarker for monitoring functional OS changes during metastasis.

## Background

Osteosarcoma (OS) is the most common primary bone tumor, with most new cases diagnosed in the first two decades of life [1, 2]. OS has a high propensity for metastasis, and over 90% of OS metastases are to the lungs [3]. The 5‑year survival of patients with primary OS is about 70%. However, the survival of metastatic OS has been below 30% for several decades, despite the introduction of multi-agent chemotherapy [4]. Much of this stagnation relates to our continued lack of understanding of the mechanisms of how OS cells migrate to the lungs, and what properties of the lung micro-environment are ideal for OS development and proliferation. Understanding the metastatic milieu is important to the development of novel strategies to inhibit OS metastases, and may lead to improved treatment outcomes for patients who already have metastatic OS.

It is believed that OS cells flourish in the lung microenvironment because of its high degree of vascularization and oxygenation. However, the pulmonary microenvironment is not unique in these traits. Prior work suggests that the specific interaction between OS and lung cells is the major determinant of three general fates of metastatic cells: proliferation, quiescence or apoptosis. However, our ability to regulate the OS-microenvironment to direct cells to anti- or pro-metastatic outcomes remains limited.

Here we describe an in vitro lung and OS cell co-culture model to explore the interactions between these cell types. We hypothesize that lung cells promote OS cell migration and survival.

## Methods

### Cell lines and culture

Three human osteosarcoma (OS) cell lines, SJSA-1, Saos-2, and U-2 OS were purchased from American Type Culture Collection (ATCC) and cultured according to ATCC’s recommendations. Two human lung cell lines, HULEC-5a and MRC-5, were also purchased from ATCC and cultured according to their recommendations.

### OS cell migration and proliferation

HULEC-5a and MRC-5 lines were cultured in growth medium for 72 h. The conditioned media (CM) was collected and centrifuged at 2000 rpm for 5 min. Each well of a 24-well plate received 600 μl CM. A total of 1x10^4^ OS cells were re-suspended in 100 μl basic DMEM (without supplements), and loaded into a Corning Transwell permeable support with a pore size of 8 μm, which was then placed into each well of the dish. After 48 h, the Transwell supports were removed and the migrated cells were quantified. CM from the murine fibroblast cell line NIH-3 T3 and fresh growth medium were utilized as controls. Proliferation was measured by co-culturing cells for 72 h, then removing the Transwell support and changing the media to standard growth medium. Cells were allowed to proliferate for 10 days, then fixed and Giemsa stained.

### Real-Time OS cell migration and invasion

HULEC-5a and MRC-5 CM were harvested as above. Utilizing the xCelligence Real-Time Cell Analysis system (Acea Biosciences, Inc., San Diego, CA) real-time cell migration was measured using CIM-plates seeded with 2x10^4^ OS cells in 100 μl of serum free media. Readings were taken every 15 min for 100 cycles and cell index was plotted at different time points. The Cell Index is defined as (Rn-Rb)/15, where Rn is the cell-electrode impedance of the well when it contains cells and Rb is the background impedance of the well with the media alone. OS cell invasion assays were created in a similar manner, with 3% Corning Matrigel used to pre-coat the upper chamber.

### OS and lung direct co-culture

OS cells were stably transduced with a lentivirus encoding for green fluorescent protein (GFP). A total of 2x10^4^ OS_GFP_ cells were seeded in a 24-well plate. These same wells were seeded with either 2x10^4^ HULEC-5a or MRC-5 cells. After 72 h, cell morphology was visualized by fluorescence microscope.

### Alkaline phosphatase (ALP) staining

A co-culture of 2x10^4^ OS cells and 2x10^4^ HULEC-5a was performed for 24, 48 and 72 h. Multiple time points were included to determine if ALP expression was different between each group at a given time. At each time point, cells were fixed and stained for ALP expression using Sigma*FAST* BCIP/NBT (Sigma-Aldrich Co LLC, USA). Similar to the direct OS and lung cell co-culture, OS cells were also cultured in CM from HULEC-5a for 72 h and stained for ALP.

### Real-time PCR

SJSA-1 and Saos-2 cells (1x10^5^ each) were cultured in growth media or HULEC-5a CM for 48 h. Total RNA was harvested using Ambion Trizol Reagent (ThermoFisher Scientific, USA). RNA (1 μg) was utilized for cDNA using Applied Biosystems High Capacity cDNA kit (ThermoFisher Scientific, USA). A total of 8 ng of cDNA was used as template and PCR was run on an Applied Biosystems StepOne Real-Time PCR Thermocycler (ThermoFisher Scientific, USA). ALDH1 primer sequence was forward: 5’-CCTGTCCTACTCACCGATTTG-3’ and reverse: 5’-CCTCCTCAGTTGCAGGATTAAA-3’.

### Disulfiram treatment

Disulfiram (Sigma-Aldrich Co LLC, USA) was dissolved in DMSO and in working concentrations of 10, 50, 100, 200 and 500 nM in growth medium or HULEC-5a CM. In the CM culture group, 2x10^4^ SJSA-1 or Saos-2 cells were seeded in each well of a 24-well plate for 24 h. In the co-culture group, 2x10^4^ HULEC cells together with 2x10^4^ SJSA-1 or Saos-2 cells were seeded in each well of a 24-well plate for 24 h. This was followed by adding fresh growth media containing disulfiram and culturing for another 72 h. Cells were then fixed and stained for ALP.

### 5-Bromo-2’-deoxyuridine (BrdU) staining

A 10 mM stock solution of BrdU (Sigma-Aldrich Co LLC, USA) was diluted 1:1000 in growth medium or HULEC CM. SJSA-1 or Saos-2 cells (2x10^4^) were seeded in a 24-well plate for 24 h. Cell medium was changed to BrdU-containing medium for another 4 h. A BrdU staining kit was used for immunohistochemistry (ThermoFisher Scientific, USA).

### Terminal deoxynucleotidyl transferase-mediated dUTP nick end-labeling (TUNEL) assay

OS Cells were grown in 24-well plates at a seeding density of 2x10^4^cells-per-well in growth media or HULEC-CM for 48 h. TUNEL assay was carried out using ApoptTag Peroxidase In Situ Apoptosis Detection Kit (EMD Millipore, Billerica, MA, USA).

### Statistical analysis

Data was analyzed using Prism 7.0 (GraphPad, La Jolla, CA, USA). Multi-group analysis was performed using analysis of variance with Tukey’s post-test for between-group comparisons. Two-group analysis was performed using *t*-tests for parametric data and the Mann-Whitney *U* test for non-parametric distributions. In all cases, *p* <0.05 was considered significant. Values were expressed as mean ± standard deviation.

## Results

### Lung cell conditioned medium (CM) induces OS cells migration

**Fig. 1:**
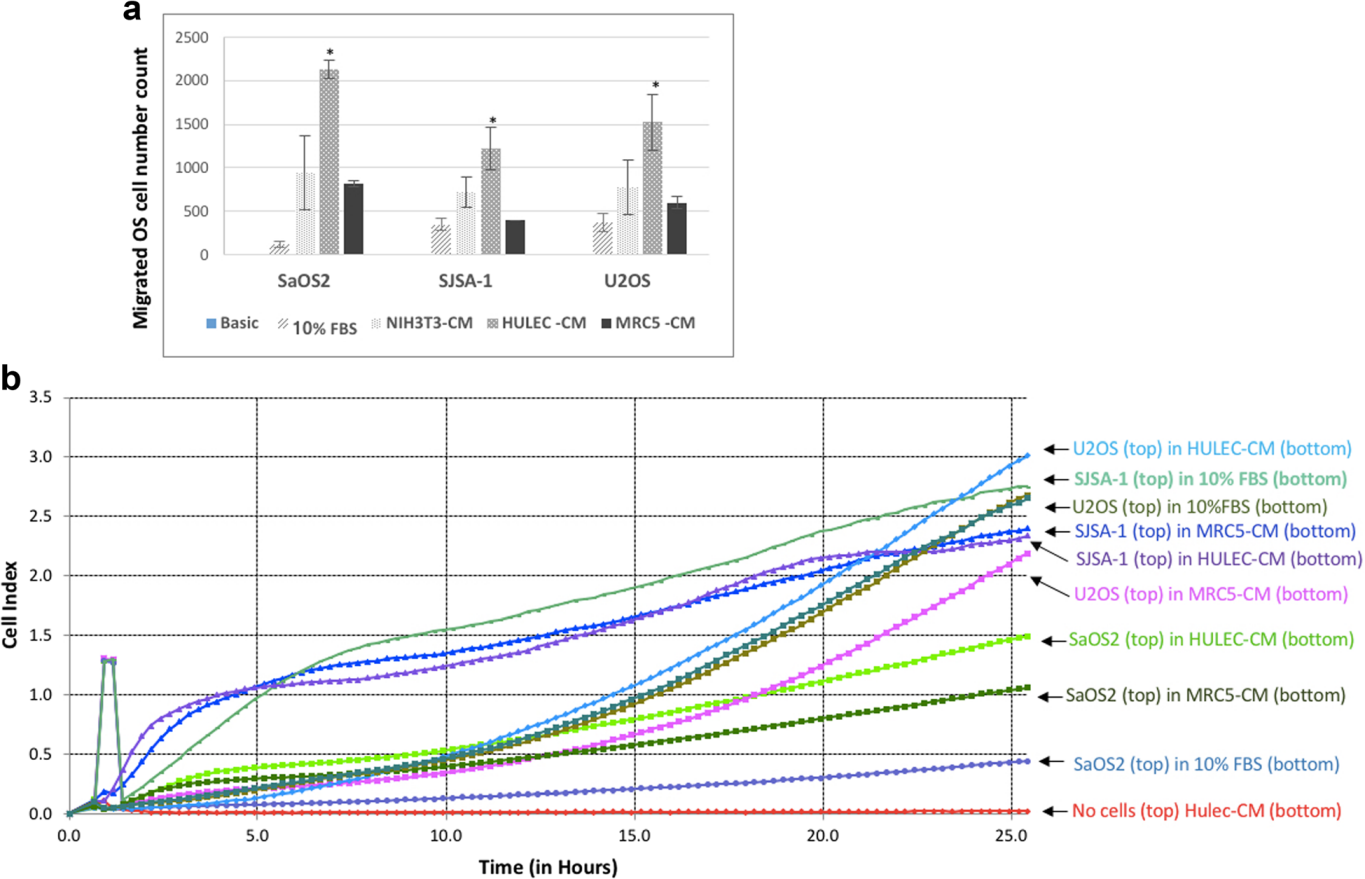
Osteosarcoma cell migration increased in lung cell conditioned medium (CM). **a** Osteosarcoma cells migration in HULEC-5a and MRC5 cell CM using transwell co-culture, NIH3T3 cell CM and cell culture medium (containing 10% FBS) are used as control. 600 μl CM was loaded to bottom chamber of 24-well plate and 2x10^4^ SaOS2, SJSA-1 and U2OS cells in 100 μl serum free medium were added to the top insert of transwell. Top insert containing OS cells was put back were cultured in CM for 48 h, followed by counting the number of migrated cells. **b** Osteosarcoma cells migration in HULEC-5A and MRC5 cell CM using xCELLigence system

To evaluate if different types of lung cells are variably attractive to different OS cell lines, we used three OS cell lines: Saos-2, SJSA-1 and U-2 OS, and two lung cell lines, HULEC-5a and MRC-5, to perform Transwell experiments. After 48 h, HULEC-5a CM had a significantly higher (*p* <0.05) attractive effect on all three OS cell lines compared to basic growth medium, 10% serum containing medium and NIH3T3 CM. Among these three OS cell-lines, Saos-2 cell had the highest and SJS1-1 the lowest migration in HULEC-5a CM (Fig. 1a). MRC-5 CM was more attractive to all three OS cell lines compared to basic medium and 10% serum containing medium, but not to NIH3T3 CM (Fig. 1a). To further evaluate the dynamic migration of these three OS cell lines in HULEC-5a CM and MRC-5 CM, we repeated the migration experiment using the xCelligence Real-Time Cell Analysis system. HULEC-5a CM had a significantly higher attractive effect on all three OS cell lines. Moreover, real-time migration data reveals that SJSA-1 cell migration in HULEC-5a CM peaks at 5 h, U-2 OS cell migration peaks at 15 h and Saos-2 migration peaks at 20 h (Fig. 1b). Migration peaks were determined by assessing the point at which the slope of the curve was greatest.

### Lung cells further increase OS cells migration

**Fig. 2:**
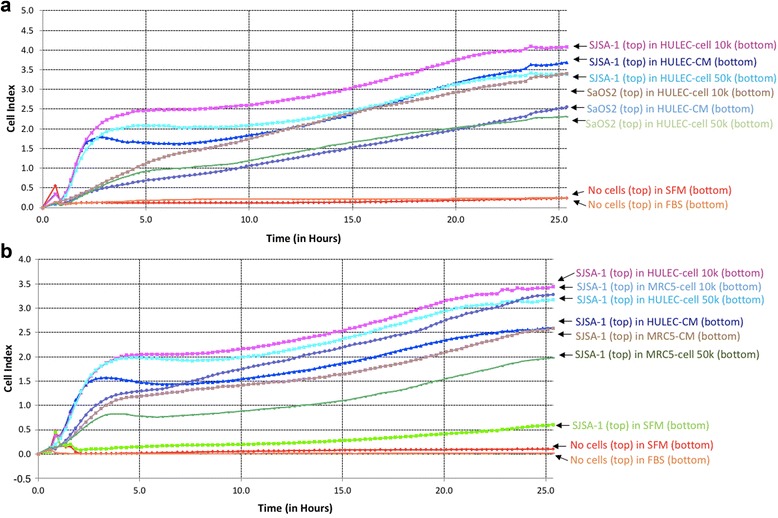
Osteosarcoma cells migration to lung cell CM were further increased in the presence of lung cells that were placed in the bottom chamber of the transwell. **a** 160 μl of HULEC-5a CM, HULEC-5a CM containing 1x10^4^ HULEC cell, and HULEC-5a CM containing 5x10^4^ HULEC cell were loaded onto the bottom well of CIM Plate 16. 2x10^4^ SaOS2 or SJSA-1 cells in 100 μl serum free medium were added to the top well of CIM Plate 16. OS cell migration index was calculated every 15 min for 100 reading. Each plate had negative controls where no cells were added to the top well and either serum free media or FBS was added to the bottom well. **b** 160 μl of HULEC-5a CM, HULEC-5a CM containing 1x10^4^ HULEC cell, and HULEC-5a CM containing 5x10^4^ HULEC cell were placed in the bottom chamber of the transwell and compared to MRC5 CM, MRC5 CM containing 1x10^4^ MRC5 cell and MRC5 CM containing 5x10^4^ MRC5 loaded onto the bottom well of CIM-Plate 16. 2x10^4^ SJSA-1 cells in 100 μl serum free medium were added to top well of CIM Plate 16, and SJSA-1 cell migration index was calculated every 15 min for 100 reading. Each plate had negative controls where no cells were added to the top well and either serum free media or FBS was added to the bottom well. Serum free media in the bottom well was also tested with SJSA cells

To dissect the role of lung cells in the attraction of OS cell migration, we investigated Saos-2 and SJSA-1 cells migration in HULEC-5a CM containing 1x10^4^ or 5x10^4^ HULEC cells. We found that 1x10^4^ and 5x10^4^ HULEC-5a cells, when in the bottom well, increased SJSA-1 cell migration compared to HULEC-5a CM in the bottom well. Interestingly, Saos-2 migration in 1x10^4^ HULEC-5a cells is significantly higher than in 5x10^4^ HULEC-5a cells or CM (Fig. 2a). SJSA-1 cell migration started earlier than Saos-2 in both HULEC-5a cells and CM (Fig. 2a). When evaluating SJSA-1 migration in MRC-5 cells and CM, we found that 1x10^4^ MRC-5 cells led to SJSA-1 cell migration, whereas MRC-5 CM and 5x10^4^ cells had lower migration rates than 1X10^4^ cells (Fig. 2b).

### Lung cell CM stimulates OS cell morphology change and invasion

**Fig. 3:**
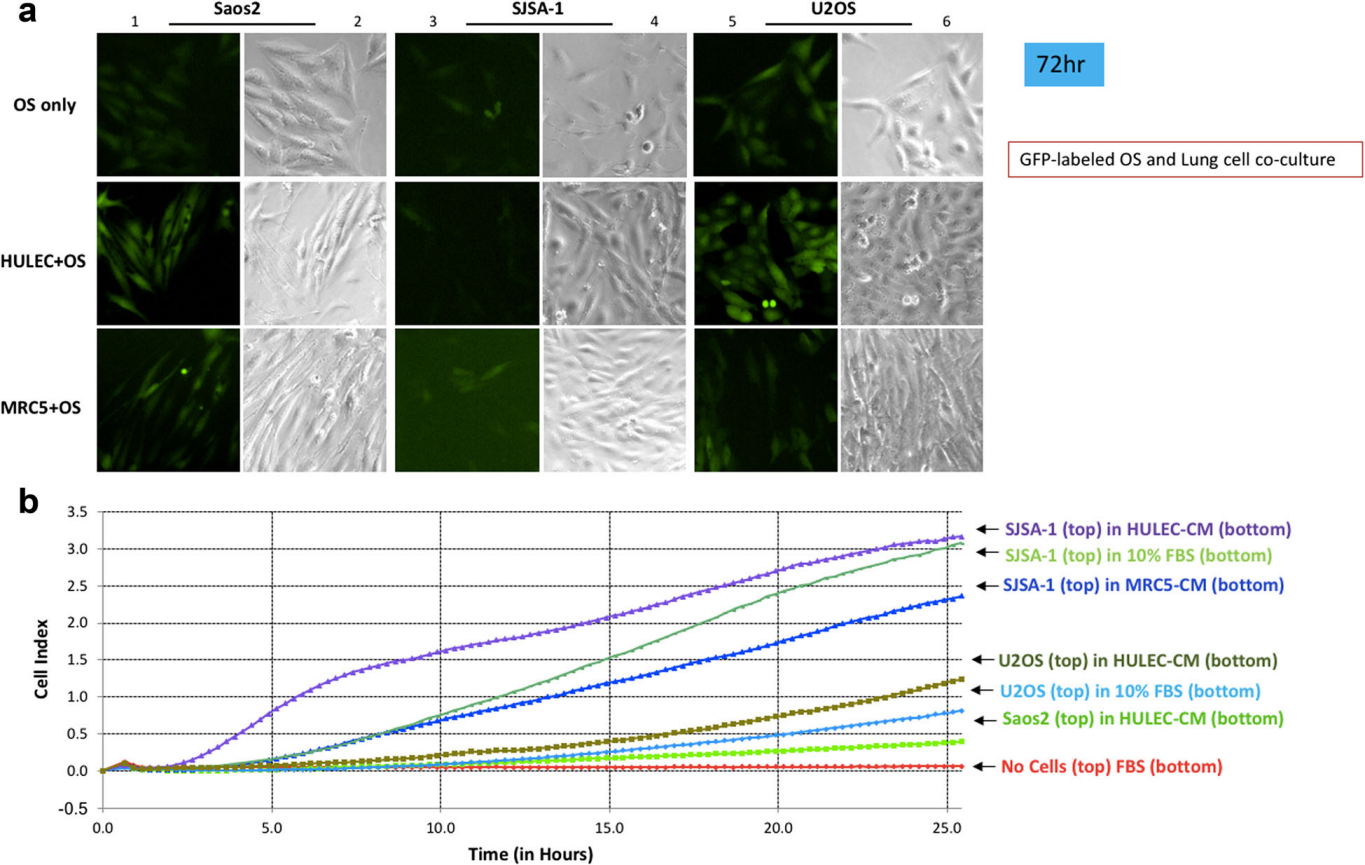
Osteosarcoma cells morphology changed in the co-culture of HULEC-5a and MRC5 cell. **a** GFP-labeled SaOS2, SJSA-1 and U2OS cells were co-cultured with HULEC-5a or MRC5 cells for 72 h. **b** Osteosarcoma cells invasion to in HULEC-5A and MRC5 cell CM using xCELLigence system. Top wells of CIM-plate 16 were pre-coated by 3% Matrigel and incubated in 37 °C for 4 h, followed by performing the same procedure in cell migration in Fig. 2

We used a lentivirus encoding for GFP to label the Saos-2, SJSA-1 and U-2 OS cell lines (Fig. 3a, column 1, 3, 5). Bright field pictures were taken of the same area (Fig. 3a, column 2, 4, 6). We used several parameters to assess morphologic changes in OS cells in the OS and lung cell co-culture. Gross morphologic difference is measured by a systematic difference in two-dimensional projected area. Our results showed that Saos-2 and SJSA-1 cells have a significantly larger projected area when in co-culture with HULEC-5a or MRC-5 cells compared to isolated Saos-2 and SJSA-1 culture alone (Fig. 3a, columns 1 and 3). U-2 OS cells demonstrated the opposite trend, and had a significantly smaller projected area in co-culture with HULEC-5a and MRC-5 cells compared to U-2 OS cells alone (Fig. 3a, column 5). Cell shape assesses roundness vs elongation. Similar to changes in projected area, Saos-2 and SJSA-1 cells showed greater elongation and less roundness when in co-culture with HULEC-5a or MRC-5 cells compared to isolated Saos-2 and SJSA-1 culture (Fig. 3a, columns 1 and 3). U-2 OS again displayed the opposite trend, showing reduced elongation but more roundness when in co-culture with HULEC-5a and SJSA-1 cells compared to U-2 OS alone (Fig. 3a, column 5).

Considering the cytoskeletal morphologic changes during the acquisition of OS invasive capacity [5–7], we assessed OS invasion in 3% Matrigel to HULEC-5a CM and MRC-5 CM, again with the xCELLigence Real-Time Cell Analysis system. SJSA-1 had a significantly higher invasion capacity in the presence of HULEC-5a CM. In contrast, U-2 OS cell invasiveness was relatively lower, and Saos-2 cell migration was completely blocked by Matrigel (Fig. 3b).

### Lung cells induce ALP expression in OS cell lines

**Fig. 4:**
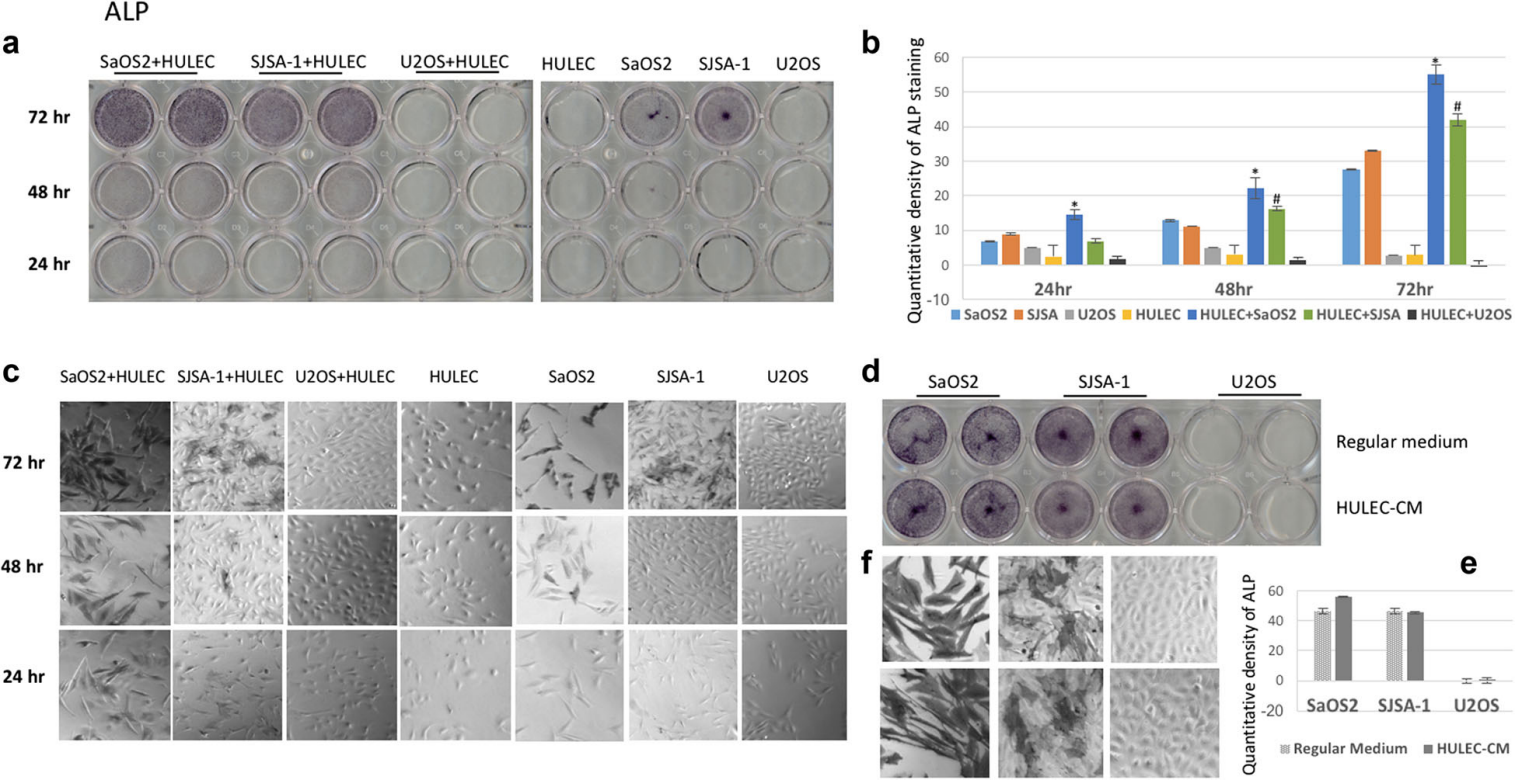
Osteosarcoma cell ALP expression increased in the co-culture of HULEC-5a cell. **a** SaOS2, SJSA-1 and U2OS cells were co-cultured with HULEC-5a cells for 24, 48 and 72 h, followed by ALP staining assay. Single cell type culture of SaOS2, SJSA-1, U2OS and HULEC-5a were used as control. **b** Quantitative densitometry assays for ALP staining in Fig. 4a. **c** High magnitude (100x) photos of ALP staining in Fig. 4a. **d** SaOS2, SJSA-1 and U2OS cells were cultured in regular medium or HULEC-5a CM for 72 h, followed by ALP staining. **e** Quantitative densitometry assays for ALP staining in Fig. 4d. * indicates the significant change of the ALP expression between SaOS2 and SaOS2 and HULEC-5a cell co-culture; # indicates the significant change of the ALP expression between SJSA-1 and SJSA-1 and HULEC-5a cell co-culture. **f**) High magnitude (100x) photos of ALP staining in Fig. 4d

Alkaline Phosphatase (ALP) has been long appreciated to correlate with active bone metabolism, and high ALP levels correlate with OS metastases [8–11]. In addition to morphologic changes, the OS metastatic phenotype correlates with alkaline phosphatase (ALP) staining in OS cells that have also changed in the presence of HULEC-5a co-culture. ALP-positive staining appeared after 24 –48 h in Saos-2 and SJSA-1 co-culture with HULEC-5a, while ALP expression in Saos-2 and SJSA-1 cells was undetectable at 24 h and slightly increased at 48 h (Fig. 4a, b, c). At 72 h, ALP staining in Saos-2 and SJSA-1 in co-culture with HULEC-5a was still significantly greater than from Saos-2 or SJSA-1 cells alone (Fig. 4a, b, c). ALP in U-2 OS and HULEC-5a cells was undetectable at 24, 48 and 72 h (Fig. 4a, b, c). To determine if HULEC-5a cells are required to increase ALP expression, we used HULEC-5a CM in the co-culture and analyzed ALP expression in Saos-2, SJSA-1 and U-2 OS. There was no significant difference in ALP expression between regular medium and CM (Fig. 4d, e, f).

### Lung cell supported migrated cell survival

**Fig. 5:**
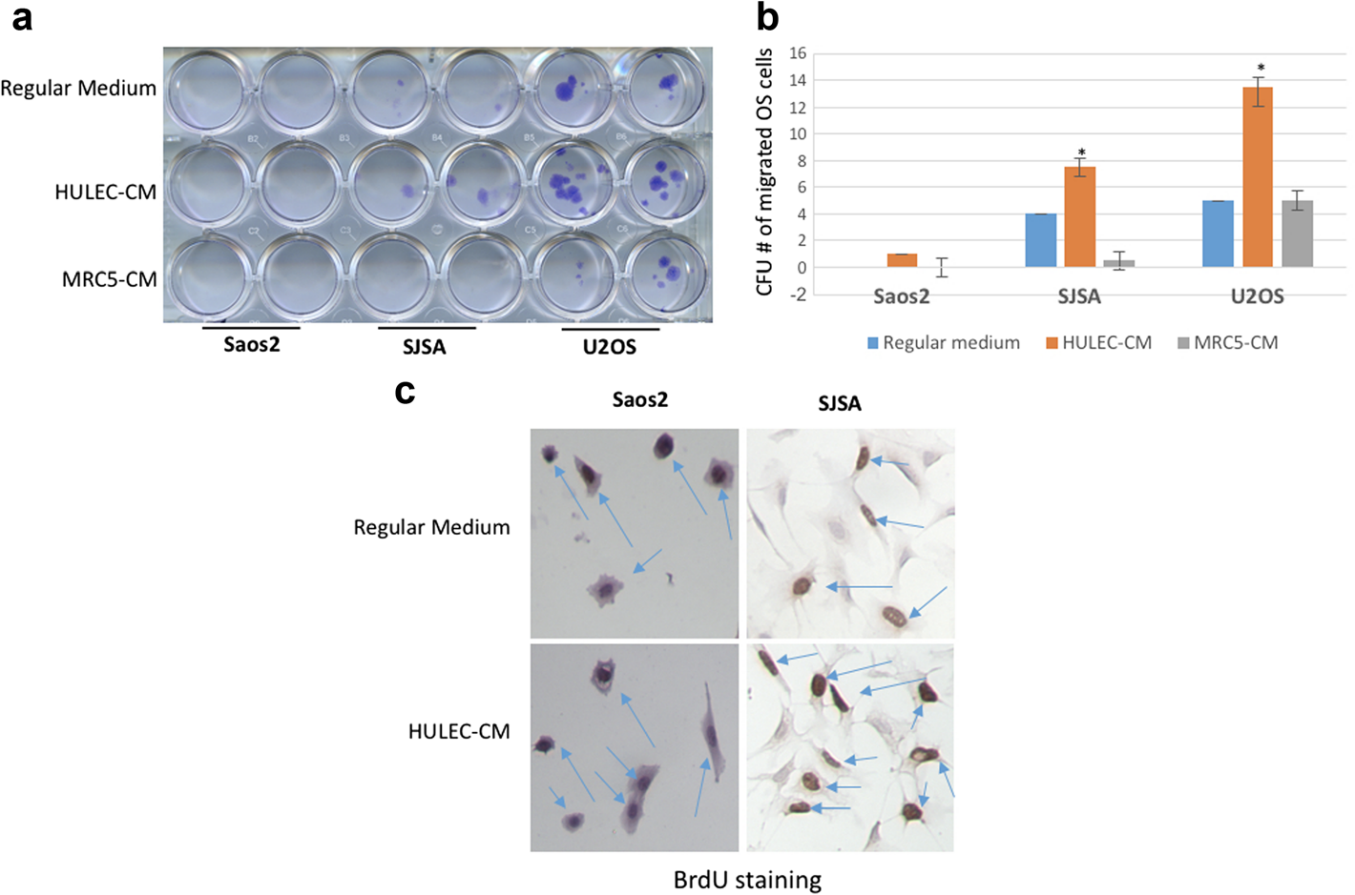
Migrated Osteosarcoma cells proliferation and survival increased in the HULEC-5a CM. **a** SaOS2, SJSA-1 and U2OS cells were induced to migrate to HULEC-5a or MRC5 CM for 48 h, followed by culturing the migrated OS cells in regular culture medium for another 10 days. OS cells were fixed and visualized by Giemsa staining. OS cell migration in regular medium is used as control. **b** Quantitative assays for Giemsa staining positive colony of the OS cell in Fig. 5a. **c** BrdU staining of the SaOS2 and SJSA-1 cells migrating to HULEC-5a and MRC5 CM. Brown color represents the positive staining

The survival of migrating OS cells is a critical step in OS metastasis. To explore the effects of lung cells on migrated OS cell survival, we induced Saos-2, SJSA-1 and U-2 OS cell migration in HULEC-5a and MRC-5 CM for 72 h, then switched to regular culture medium for an additional 10 days. Compared to migrated Saos-2 and SJSA-1 cells, migrated U-2 OS cells had the most colony growth in regular medium, HULEC-5a CM and MRC-5 CM. Migrated SJSA-1 cells grew in HULEC-5a CM, but not MRC-5 CM. Migrated Saos-2 cells were not observed in HULEC-5a CM, MRC-5 CM and control media (Fig. 5a, b). To uncover the mechanisms underlying increased cell migration, we assessed migrated OS cell proliferation and found that HULEC-5a CM benefited SJSA-1 cell proliferation (Fig. 5c).

### ALDH is involved in the process of lung: OS cell interaction

**Fig. 6:**
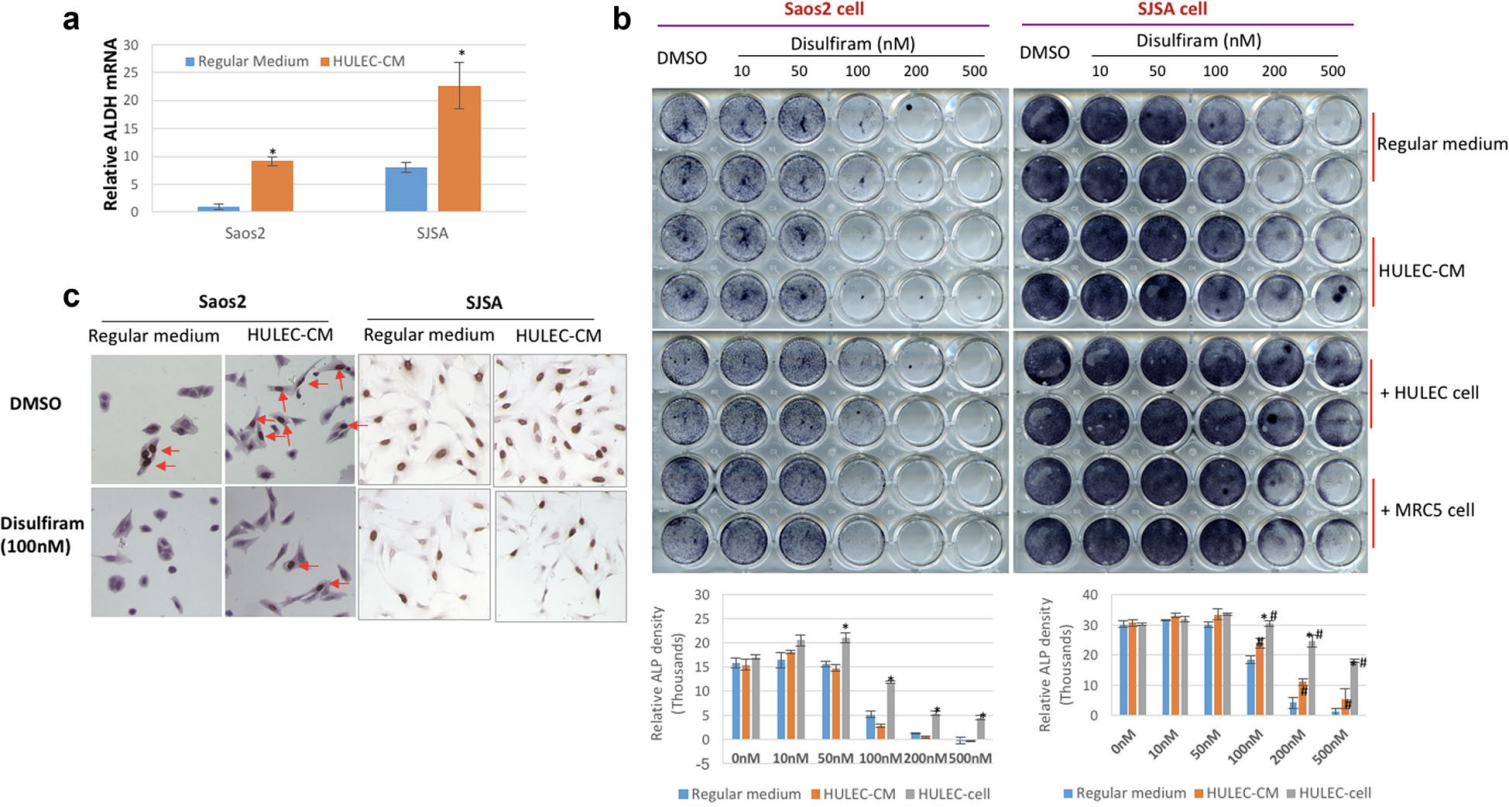
ALDH is involved in the increased migration of Osteosarcoma cells induced by HULEC-5a cell and CM. **a** ALDH mRNA level in SaOS2 and SJSA-1 cells is increased in HULEC-5a CM compared with regular medium. **b** ALP expression in SaOS2 and SJSA-1 cell was suppressed by ALDH inhibitor disulfiram in a dose-dependent manner, and HULEC-5a cell and CM rescue the ALP expression which is inhibited by disulfiram. Quantitative densitometry assays for ALP staining in Fig. 6b. * indicates the significant difference of the ALP expression in SaOS2 and SJSA-1 cells between single cell type culture of SaOS2/or SJSA-1 and the co-culture of SaOS2 and HULEC-5a cell treated with indicated dose of disulfiram. # indicates the significant difference of the ALP expression between regular medium and HULEC-5a CM in SJSA-1 cells treated with indicated dose of disulfiram. **c** BrdU staining for the SaOS2 and SJSA-1 cells treated with/without disulfiram. Brown color represents the positive staining

Akdehyde dehydrogenase (ALDH) mRNA level in Saos-2 and SJSA-1 cells was elevated after being cultured in HULEC-5a CM, at a fold change of 9 and 22, respectively (Fig. 6a). To further investigate the role of this cancer stem cell marker in the interaction between lung and OS cells, we treated co-cultured cells with disulfiram, an ALDH-specific inhibitor, at doses of 10, 50, 100, 200 and 500 nM for 72 h. We found that ALP staining of Saos-2 cells was significantly suppressed in a dose dependent manner, in particular at a dose of 100 nM and higher. Moreover, HULEC-5a cells reversed the disulfiram-mediated suppression of ALP in Saos-2 cells at 100 nM, but not at higher disulfiram doses of 200 and 500 nM (Fig. 6b). HULEC-5a CM did not affect the disulfiram-mediated suppression of ALP in Saos-2 cells (Fig. 6b). ALP expression of SJSA-1 was also significantly inhibited by disulfiram in a dose-dependent manner. HULEC-5a cells attenuated the suppression of ALP expression in SJSA-1 cells even at higher doses of disulfiram (500nM). The effect of HULEC-5a CM on the ALP expression of SJSA-1 cells is similar to HULEC-5a cells (Fig. 6b). On the contrary, MRC-5 cells did not influence the disulfiram-mediated inhibition of ALP expression in Saos-2 and SJSA-1 cells (Fig. 6b, d). Interestingly, HULEC-5a CM increases Saos-2 and SJSA-1 cell proliferation in the presence of 100 nM disulfiram for 48 h (Fig. 6c).

## Discussion

OS is the most common primary malignant bone tumor, with peak incidence rates during adolescence [12–14]. Pulmonary OS metastasis is the predominant determinant of disease mortality [4]. However, the mechanisms underlying OS cell migration to the lungs and their interaction with lung cells remain poorly understood. Here we found that different OS cell sub-types have different capacities for migration in response to the same human lung cell-line. The SJSA-1 cell-line, a so-called fibroblastic OS cell-line with a high metastatic potential, had significantly greater migration and metastasis marker gene expression in response to HULEC-5a cell exposure compared to Saos-2 and U-2 OS cells, two epithelial OS cell-lines. Further, different types of lung cells have different attractive potential. SJSA-1 migration and invasion in the presence of HULEC-5a, a lung endothelial cell-line, is significantly greater than in the presence of MRC-5, a fibroblastic lung cell-line.

### Heterogeneity of OS cell with different metastatic potential has different response to lung cell environment: cell or cell-conditioned medium

The SJSA-1 cell line has been used as an OS metastatic model in several prior studies [15–19], and Saos-2 and U-2 OS cells have been found to have an undetectable metastatic effect [16, 19]. We therefore used SJSA-1 as our pro-metastatic cell model, and Saos-2 and U-2 OS as controls. In line with previous reports, our data demonstrated that SJSA-1 has a significantly higher migration potential when exposed to HULEC-5a CM compared to control medium (Figs. 1a, b, 2a, b, 5a and b). Although Saos-2 cells have a slightly higher migration rate than SJSA-1 cells (Fig. 1b), Saos-2 cells cannot survive in a novel microenvironment (Fig. 5a and b). U-2 OS cells have similar migration and survival capabilities to SJSA-1 cells when exposed to HULEC-5a CM (Figs. 1a, 5a and b). However, U-2 OS cells rarely invade through the basement membrane of distant tissue compared to SJSA-1 cells (Fig. 3b). It is well-accepted that cancer metastasis involves several critical stages, including tumor invasion through the local basement membrane, tumor cell survival within the bloodstream and the initial survival of tumor cells in the distant tumor stromal environment [20, 21]. SJSA-1 has dramatically higher invasion (Fig. 3b), migration (Fig. 1a and b), survival and proliferation potential in lung cell co-culture (Fig. 5a and b). This reflects the high metastatic potential of SJSA-1 in animal models.

An interesting finding in our study was the different invasion and migration potential of OS cells following lung cell co-culture. SJSA-1 had a higher invasiveness (Fig. 3b) but relatively lower migration potential (Fig. 1b) compared to cells grown in HULEC-5a CM. Saos-2 and U-2 OS have higher migration (Fig. 1a), but reduced invasion potential compared to cells grown in HULEC-5a CM (Fig. 3b). To migrate, the cell body must modify its shape and stiffness to interact with the surrounding tissue structures. Invasion requires adhesion, proteolysis of extracellular matrix components and migration [22]. SJSA-1 cells become polarized and elongate when co-cultured with HULEC-5a cells (Fig. 3a), an alteration that may be a sentinel event of cell invasion. In contrast, U-2 OS cell morphology does not change when co-cultured with HULEC-5a cells (Fig. 3a).

### Different lung cell types have different effects on OS cell migration

Lung tissue is made up of multiple cell types, including epithelial, endothelial and fibroblastic cells. To characterize which cell type most effectively attracts OS cells, we used CM from HULEC-5a, an endothelial cell-line, and MRC-5, a fibroblastic cell-line. Our data showed that HULEC-5a CM is a more efficient attractant for OS cell migration compared to MRC-5 CM (Figs. 1b, b, 2a and b).

To further investigate if the lung cells themselves are required to stimulate OS migration, we used CM containing doses of HULEC-5a cells or MRC-5 cells. Our data demonstrate that 1x10^4^ HULEC-5a or MRC-5 cells further stimulate SJSA-1 cell migration compared to HULEC-5a CM alone (Fig. 2a and b). This suggests that some unknown properties of HULEC-5a and MRC-5 cells also play important roles in the promotion of OS cell migration. Interestingly, 5x10^4^ HULEC-5a or MRC-5 cells have the same effect as CM controls (Fig. 2a and b). The reason why higher doses of HULEC-5a and MRC-5 cells have a reduced effect on SJSA-1 cell migration remains unclear. One possible explanation may be because too many cells are competing for available nutrients in vitro and producing cytotoxic byproducts.

### ALP activity is a marker for OS lung metastasis

ALP has been previously demonstrated to be an indicator of OS metastasis in multiple clinical studies [8–11]. High serum ALP levels are associated with the presence of metastatic OS at the time of diagnosis, and poor disease prognosis. Serum ALP level is a convenient and effective biomarker of OS prognosis. In our study we used ALP expression to monitor OS cell functional changes in response to culture with HULEC-5a cells or CM. We found that ALP expression in SJSA-1 and Saos-2 cells was dramatically increased by HULEC-5a cell co-culture. ALP expression was also noted at earlier time points (Fig. 4a, b and c). In contrast, ALP expression was not influenced by HULEC-5a CM (Fig. 4d, e and f). This suggests that functional changes to OS cells requires direct interaction with lung cells.

### ALDH is involved in the process of OS cell migration to lung cell

ALDH is a biomarker for cancer stem cells [23, 24]. Previous studies by our group have shown that ALDH activity is greater in the highly metastatic K7M2 OS cell-line than in non-metastatic K12 OS cells. We have also correlated ALDH activity with clinical OS metastasis, suggesting that ALDH may be a therapeutic target specific to OS cells with high metastatic potential [25]. Subsequent studies found that Retinal, the precursor to retinoic acid with known antitumor properties, targets ALDH-positive cancer stem cells and alters the phenotype of highly metastatic OS cells [26]. When determining if ALDH was involved in OS cell functional changes in response to the HULEC-5a microenvironment, we found that HULEC-5a CM dramatically increases ALDH expression in SJSA-1 and Saos-2 cells (Fig. 6a). In contrast, in our loss-of-function experiments we treated our experimental groups with doses of disulfiram, an ALDH inhibitor. Our findings demonstrated that HULEC-5a cells help SJSA-1 to resist disulfiram inhibition and maintain their pro-metastatic function, indicated by their preserved ALP activity (Fig. 6b). HULEC-5a cells also supported SJSA-1 cell survival and proliferation in the presence of disulfiram (Fig. 6c). Our current results are consistent with our previous findings [25, 26] as well as those of other groups [23, 24].

While the findings of this study contribute to our understanding of the mechanisms behind OS metastasis, we recognize several limitations to this work. Our sample size throughout this study was often based on single experiments, which highlights the preliminary nature of our work. Our utilization of a murine fibroblast cell line as a control was a decision made out of convenience, as our group has significant experience and success with this cell line. This will be altered in future studies. However, we believe that the relationships highlighted here would persist regardless of cell line used.

Most importantly, our findings illustrate associative relationships between OS cells and lung cells. While these relationships may serve as the basis for new theories, we have not established mechanisms (beyond ALDH activity) that explain them. While we concede that our findings do not yield mechanistic conclusions, we believe that the data presented here (1) describe a new strategy by which the mechanisms behind OS metastasis may be elucidated, and (2) suggest that lung cells per se are active participants in the metastatic process, either directly or indirectly or both. Heretofore, this relationship has been suggested by OS’s clinical behavior, but not demonstrated. We hope that our findings will serve as the foundation of future work that will identify causal factors and mechanisms that will explain the provocative phenomena we report here.

## Conclusions

We have demonstrated that fibroblastic SJSA-1 OS cells have a higher lung metastatic potential than epithelial Saos-2 and U-2 OS cells. Lung endothelial HULEC-5a cells are attractants for OS cell migration, proliferation and survival. Further, the cancer stem cell marker ALDH is involved in the interaction between lung and OS cells, and ALP could be a valuable biomarker for monitoring functional OS changes during the metastatic process. These data will form the basis of future work that will delve deeper into understanding the mechanisms by which metastatic OS cells and the lung microenvironment interact in the metastatic process.

## Abbreviations

ALDH: Aldehyde dehydrogenase
ALP: Alkaline phosphatase
ATCC: American type culture collection
BrdU: 5-Bromo-2’-deoxyuridine
CM: Conditioned media
GFP: Green fluorescent protein
OS: Osteosarcoma
TUNEL: Terminal deoxynucleotidyl transferase-mediated dUTP nick end-labeling
